# HRQoL assessment by SRS-30 for Chinese patients with surgery for Adolescent Idiopathic Scoliosis (AIS)

**DOI:** 10.1186/1748-7161-10-S1-P31

**Published:** 2015-01-19

**Authors:** Bobby Kin Wah Ng, Wai Wang Chau, Anna Chak Na Hui, Po Yin Cheng, Chau Yuet Wong, Bin Wang, Jack Chun Yiu Cheng, Tsz Ping Lam

**Affiliations:** 1 Department of Orthopaedics and Traumatology, The Chinese University of Hong Kong, Hong Kong; 2 Joint Scoliosis Research Center of the Chinese University of Hong Kong and Nanjing University, China; 3 Department of Surgery, Prince of Wales Hospital, Hong Kong

## Objectives

SRS-30 as a health-related quality of life (HRQoL) questionnaire was established since 2003. Literatures from Asian countries on SRS-30 mainly derived from local adaptation and related validation studies. Reports on its use for measuring surgical outcomes were sparse, particularly in the Chinese community. We carried out a retrospective cohort study using SRS-30 to evaluate HRQoL for Chinese AIS adolescents before and after surgery.

## Material and methods

One hundred and four (104) Chinese AIS patients undergoing posterior spinal fusion between 2009 and 2013 were recruited. They completed SRS-30 before hospital discharge, and post-surgery questions were asked again 6 months to 3 years after discharge through phone interview. Mean scores in different domains were calculated at 3 time points, namely at the time before surgery (pre-op), immediately after surgery (discharge), and at follow-up (follow-up). Change in scores between follow-up and discharge (change after discharge) was calculated. Gender-specifc descriptive analyses were summarized. Pearson's correlations on scores collected at the 3 time points and "change after discharge" were carried out. Effects of potential risk factors (age, pre-op maximum Cobb angle, curve correction after surgery in degrees) on mean domain scores were evaluated by linear regression models.

## Results

The mean age (in years) was 17.65 (male) and 15.92 (female), and 80.8% were female. There were significant correlations between pre-op and discharge scores in function-activity (r=-0.47, p=0.05) in male. In female, correlations were found between pre-op and "change after discharge" in pain (r=-0.23, p=0.04), and satisfaction with management between pre-op and discharge (r=0.334, p<0.01) and pre-op and "change after discharge" (r=-0.47, p<0.01). Linear regression analysis showed that pre-op maximum Cobb angle was a significant predictor (B=-0.027, p=0.02) on satisfaction with management at follow-up in male patients. Comparing the scores at "change after discharge" in male showed degree of curve correction after surgery was a significant predictor in self-image-appearance (B=-0.159, p<0.01) and satisfaction with management (B=-0.123, p<0.01). Pre-op maximum Cobb angle was found to be another significant predictor (B=0.052, p=0.02) on self-image-appearance in male. In female patients, degree of curve correction after surgery was a significant predictor (B=0.045, p=0.04) on function-activity at "change after discharge".

## Conclusions

Gender differences were found of which female patients demonstrated correlations on pain and satisfaction with management before and after surgery, and male patients on function-activity. Degree of curve correction after surgery and pre-op maximum Cobb angle were significant predictors on function-activity, self-image-appearance, and satisfaction with management in AIS patients.

